# Serum Bicarbonate: Reconsidering the Importance of a Neglected Biomarker in Predicting Clinical Outcomes in Sepsis

**DOI:** 10.7759/cureus.24012

**Published:** 2022-04-10

**Authors:** Robin Paudel, Brittany Bissell, Prerna Dogra, Peter E Morris, Said Chaaban

**Affiliations:** 1 Pulmonary and Critical Care Medicine, Mayo Clinic Health System, La Crosse, USA; 2 Pharmacy Practice and Science, University of Kentucky, Lexington, USA; 3 Endocrinology (Diabetes and Metabolism), Mayo Clinic, Rochester, USA; 4 Pulmonary, Critical Care, and Sleep Medicine/Internal Medicine, University of Kentucky College of Medicine, Lexington, USA

**Keywords:** clinical outcomes, predictive value, lactate, sofa score, serum bicarbonate, sepsis

## Abstract

Background

Despite being an important pathophysiological component, information on the predictive value of serum bicarbonate level in sepsis is limited.

Study design and method

This is a single-centered retrospective study involving 4176 patients admitted to the medical ICU (MICU) with a diagnosis of sepsis. Patients were divided into two groups based on the presence or absence of chronic kidney disease (CKD) on admission: CKD and non-CKD, respectively. Each group was then divided into three sub-groups based on serum bicarbonate level at presentation (in mEq/l)- low (<22), normal (22-28), and high (>28). We compared the clinical outcomes between the sub-groups in each group, with in-hospital mortality as the primary endpoint. Secondary endpoints included vasopressor-free days, ventilator-free days, ICU-free days, and hospital-free days.

Result

In both the CKD and non-CKD groups, low serum bicarbonate was associated with significantly increased in-hospital mortality. There was no difference in the mortality between the sub-groups with normal and high serum bicarbonate. When adjusted for other known predictors of mortality, the association of low serum bicarbonate with increased in-hospital mortality was statistically significant only in the patient group with a Sequential Organ Failure Assessment (SOFA) score of ≥9. Additionally, the SOFA score had a better predictive value for in-hospital mortality, ICU-free days, and ventilator-free days when the serum bicarbonate level was <22.

Interpretation

Serum bicarbonate is a good predictor of clinical outcomes in sepsis and can be used along with other markers of sepsis to predict clinical outcomes.

## Introduction

Sepsis is a life-threatening syndrome of organ dysfunction caused by infection and dysregulated host response to the infection [1,2]. It is the leading cause of death worldwide, with an estimated incidence of 31.5 million cases of sepsis, 19.4 million cases of severe sepsis, and 5.3 million deaths per year [3]. Markers of sepsis that predict morbidity and mortality, such as Acute Physiology and Chronic Health Evaluation (APACHE) score, Sequential Organ Failure Assessment (SOFA) score, Simplified Acute Physiology Score (SAPS) score, serum lactate level, and mean arterial pressure, have been extensively studied [4-9]. Despite being an important pathophysiological component of sepsis, information on serum bicarbonate level and its predictive value in sepsis are limited. Hemodynamic instability in critically ill patients with sepsis can induce multiple organ compromise, including renal dysfunction and tissue hypoperfusion, both of which are widely believed to cause decreased serum bicarbonate levels. Recent studies have suggested that serum bicarbonate can neither be a surrogate for lactate nor can it accurately predict the lactate level in patients with sepsis [10-12]. To the best of our knowledge, ours is the first large study to look at the relation between serum bicarbonate and clinical outcomes in patients with sepsis. We hypothesize that serum bicarbonate level at presentation is a predictor of morbidity and short-term mortality in patients with sepsis admitted to the medical intensive care unit (MICU).

## Materials and methods

After approval by the University of Kentucky Institutional Review Board (approval number 47751), we performed a single-center retrospective research study on patients admitted to the MICU of the University of Kentucky Medical Center, Lexington, Kentucky, United States, with a diagnosis of sepsis, severe sepsis, and septic shock from January 2012 to December 2017. We used International Classification of Diseases (ICD) 9 and 10 codes to identify these patients. The exclusion criteria were age < 18 years, end-stage renal disease requiring dialysis, or missing serum bicarbonate level at presentation defined as within four hours of admission to MICU. A total of 5293 patients met the inclusion criteria, of which 1117 patients were excluded. The final cohort consisted of 4176 subjects. The primary outcome was in-hospital mortality, defined as death before hospital discharge. Secondary outcomes included hospital-free days, ICU-free days, ventilator-free days, and pressor-free days, all calculated at 28 days. Patients with a diagnosis of severe sepsis were included in the study because we also used ICD 9 for the identification of patients.

Stata Statistical Software: Release 13, 2013 (StataCorp LP, College Station, Texas, United States) was used for statistical analysis. We first evaluated the unadjusted predictive value of serum bicarbonate in sepsis. Then, we adjusted the outcomes for co-morbidities and other known predictors of clinical outcomes in sepsis.

For the unadjusted predictive value of serum bicarbonate, the study cohort was first divided into three groups based on serum bicarbonate level at presentation: serum bicarbonate level <22, serum bicarbonate level 22-28, and serum bicarbonate level >28. In-hospital mortality was then compared among these groups. The study cohort was then divided into two major groups based on the presence or absence of chronic kidney disease (CKD) on admission: CKD and non-CKD groups, respectively. Each group was then divided into three subgroups based on serum bicarbonate at presentation: low bicarbonate (<22 mEq/l), normal bicarbonate (22-28 mEq/l), and high bicarbonate (>28 mEq/l). The mortality in the three sub-groups within each group was then compared using a Chi-square test.

**Figure 1:**
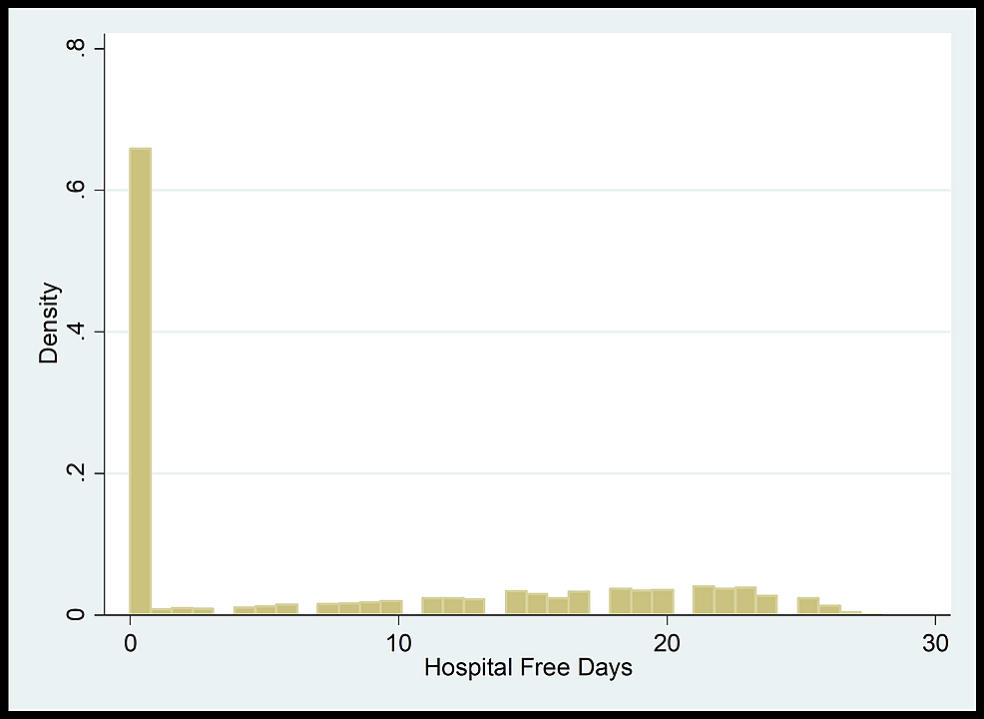
Hospital-free days distribution plot

**Figure 2:**
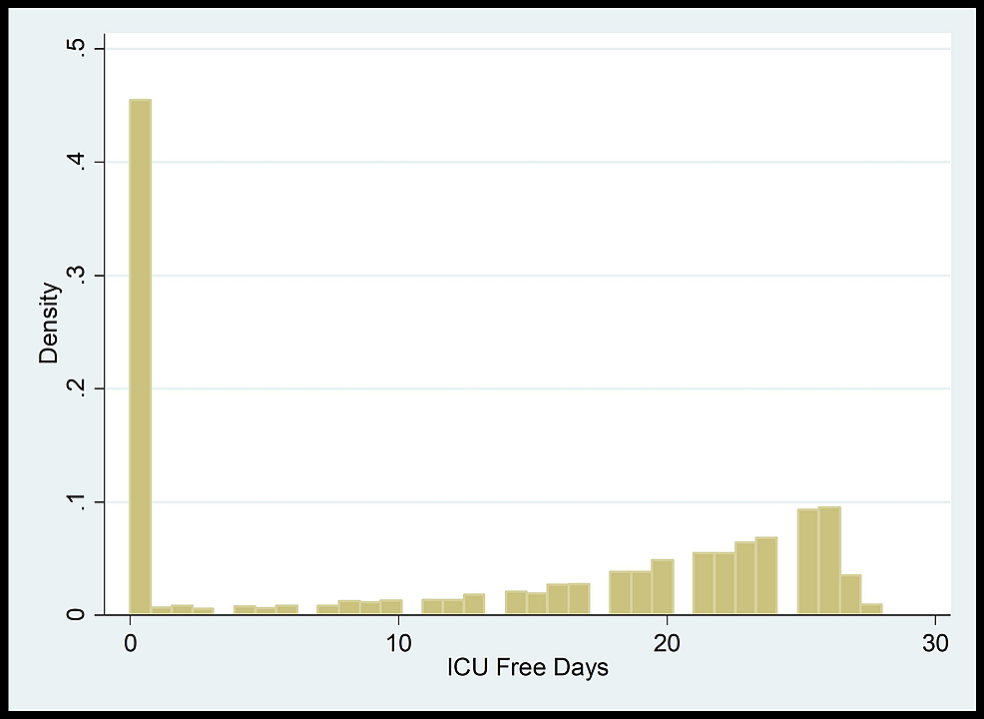
ICU-free days distribution plot

To calculate the adjusted predictive value of serum bicarbonate, the study cohort was divided into three groups: group 1 (bicarbonate level <22 mEq/L), group 2 (bicarbonate level 22-28 mEq/L), and group 3 (bicarbonate level >28 mEq/L). The clinical outcomes in groups 1 and 3 were then compared to those in group 2. We used a multiple regression model adjusted for age, gender, SOFA score, and co-morbidities (CKD, chronic obstructive pulmonary disease (COPD), liver cirrhosis). Based on the distribution shown in plots (Figure 1 and Figure 2), we conducted Zero-Inflated Poisson regression models to look at the hospital-free days and ICU-free days as clinical outcomes.

**Figure 3:**
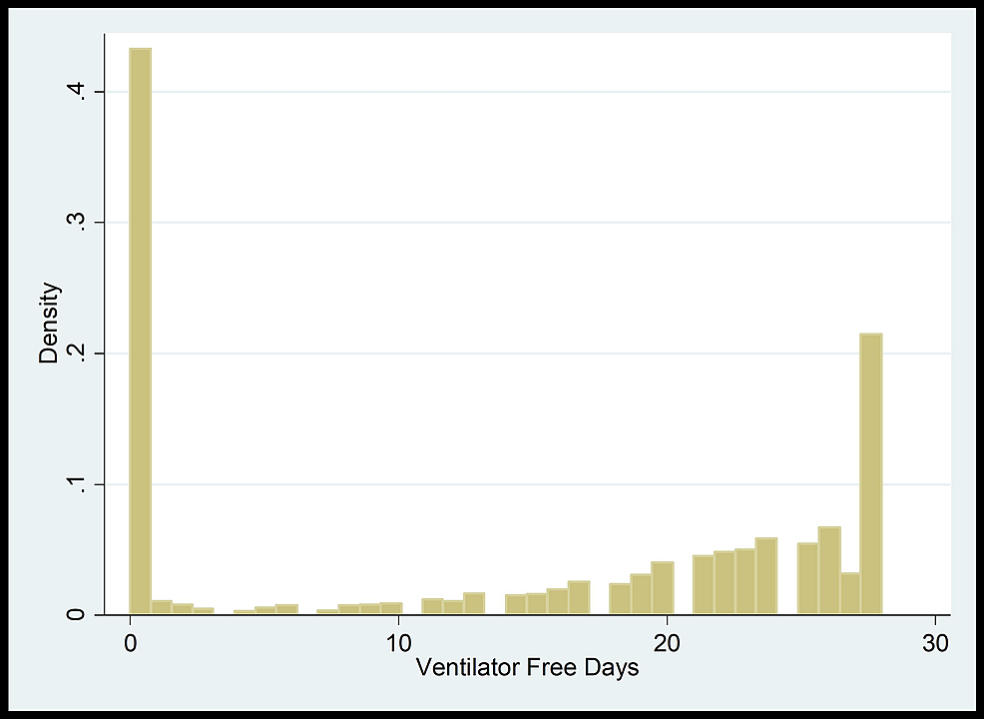
Ventilator-free days distribution plot

**Figure 4:**
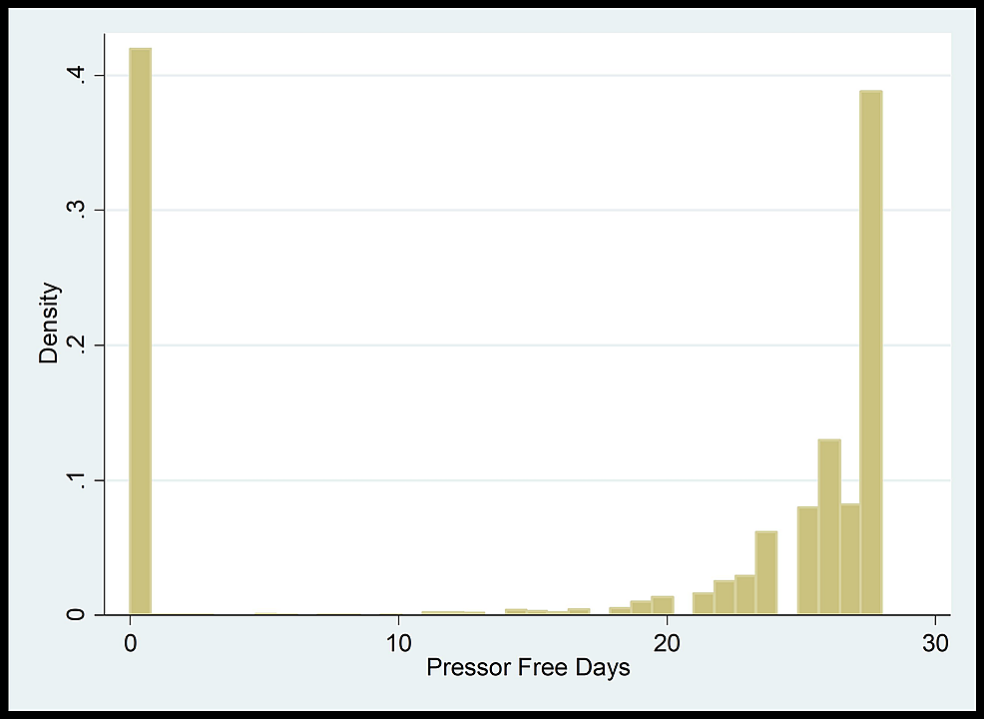
Pressor-free days distribution plot

Based on the distribution shown in plots (Figure 3 and Figure 4), we first transformed the outcome variables by dividing the ventilator-free days and pressor-free days by 28. We then conducted Zero-One Inflated Beta regression models to look at the impacts of bicarbonate on these clinical outcomes.

## Results

Patient characteristics are given in Table 1.

**Table 1:** Unadjusted for other known predictors of clinical outcomes in sepsis CKD: Chronic Kidney Disease; MICU: Medical Intensive Care Unit; BMI: Body Mass Index; SOFA: Sequential Organ Failure Assessment, eGFR: Estimated Glomerular Filtration Rate, LTACH: Long Term Acute Care Hospital; Dx: Diagnosis

	CKD Dx					No CKD Dx				
Bicarbonate on MICU Admission	< 22	22 to 28	> 28	P-value	n	< 22	22 to 28	> 28	P-value	n
Number of Patient Visits	515	246	74		835	1706	1124	511		3341
Average Age	61.911	64.053	64.932	0.010	835	53.925	54.936	57.311	< 0.001	3341
Average BMI	31.981	33.578	34.951	0.011	808	29.082	29.395	32.217	0.032	3184
Percentage Male	54.95%	56.50%	60.81%	0.622	835	48.59%	52.67%	54.01%	0.030	3341
Percentage White	91.46%	93.09%	91.89%	0.740	835	93.85%	94.22%	95.69%	0.288	3341
Average SOFA Score	10.548	8.955	7.986	< 0.001	835	10.291	7.892	7.100	< 0.001	3341
Average Heart Rate	97.480	90.069	86.919	< 0.001	835	105.370	102.713	98.638	< 0.001	3340
Average Mean Blood Pressure	72.707	77.715	79.230	< 0.001	835	73.249	79.617	81.804	< 0.001	3340
Average Temperature	97.847	98.516	98.176	<0.001	834	98.041	98.762	98.694	< 0.001	3338
Average Respiratory Rate	21.130	20.919	19.581	0.053	835	22.724	21.449	20.875	< 0.001	3339
Average Bicarbonate Minimum	13.460	20.187	25.041	<0.001	835	13.771	20.924	27.174	< 0.001	3341
Average Bicarbonate at 48 Hours	18.585	23.296	30.554	<0.001	642	19.126	24.547	31.991	< 0.001	2475
Average Lactate	3.350	1.772	1.735	<0.001	796	4.509	2.317	1.742	< 0.001	3173
Average Albumin	2.255	2.386	2.456	0.005	817	2.212	2.392	2.407	< 0.001	3262
Average Urea Nitrogen	58.277	43.646	47.486	<0.001	832	37.578	25.557	25.168	< 0.001	3336
Average Serum Creatinine	3.449	2.267	1.892	<0.001	832	2.130	1.259	0.982	< 0.001	3336
Average eGFR	24.594	37.844	46.001	<0.001	832	50.802	75.120	85.260	< 0.001	3336
Average Potassium	4.686	4.417	4.235	<0.001	834	4.367	4.126	4.169	< 0.001	3337
Average Sodium	138.217	139.594	140.608	<0.001	832	137.884	139.100	140.327	< 0.001	3337
Average Chloride	103.162	101.943	97.270	<0.001	832	102.660	101.969	98.035	< 0.001	3336
Average pH	7.240	7.339	7.385	< 0.001	745	7.265	7.363	7.382	< 0.001	3020
Average Anion Gap	19.612	15.211	13.662	< 0.001	830	19.311	14.463	12.217	< 0.001	3336
Percentage with Positive Blood Culture within 24 Hours	25.63%	13.01%	21.62%	<0.001	835	29.31%	21.44%	17.61%	<0.001	3341
Percentage with Positive Blood Culture during Visit	33.59%	27.64%	37.84%	0.144	835	38.39%	32.38%	28.57%	<0.001	3341
Average Hospital-Free Days	6.588	7.902	6.554	0.149	835	7.250	8.884	8.311	< 0.001	3341
Average MICU-Free Days	11.862	13.736	11.959	0.059	835	11.705	14.416	13.601	< 0.001	3341
Average Ventilator-Free Days	13.551	15.667	13.270	0.057	835	13.115	16.032	15.213	< 0.001	3341
Percentage Receiving Pressors	78.06%	63.01%	66.22%	<0.001	835	75.73%	56.85%	53.03%	<0.001	3341
Average Pressors-Free Days	15.732	19.122	18.743	< 0.001	835	15.441	19.537	19.959	< 0.001	3341
Percentage Discharged to Hospice	10.87%	11.38%	14.86%	0.600	835	8.68%	8.45%	7.44%	0.675	3341
Percentage Discharged Home/Home Health	19.03%	21.14%	12.16%	0.226	835	22.74%	28.83%	25.24%	0.001	3341
Percentage Discharged to Nursing Home	1.36%	4.47%	5.41%	0.013	835	2.52%	3.56%	4.11%	0.110	3341
Percentage Discharged to LTACH	6.02%	7.72%	12.16%	0.139	835	3.93%	5.60%	9.98%	<0.001	3341
In-Hospital Mortality Rate	39.81%	30.49%	33.78%	0.039	835	42.38%	29.00%	28.57%	<0.001	3341

In the final cohort of 4176 patients, irrespective of the presence or absence of CKD (Table 2), in-hospital mortality was significantly higher in the patients with low serum bicarbonate compared to normal or high serum bicarbonate (p-value<0.0001). Additionally, noted in the low bicarbonate subgroup of both the CKD and non-CKD groups were significantly high SOFA score, low mean arterial pressure, low temperature, low pH, low albumin, high lactate, low estimated glomerular filtration rate (eGFR), and high anion gap. (Table 1 ).

**Table 2:** Mortality comparison among different bicarbonate groups MICU: Medical Intensive Care Unit

Bicarbonate on MICU admission	<22	22-28	>28	p-value			
Number of patient visits (n)	2221	1370	585				
In-Hospital mortality	41.78%	29.27%	29.23%	<0.001			
Mortality in group with bicarbonate <22 compared to group with bicarbonate 22-28, chi2=56.9093 (p-value <0.0001)							
Mortality in group with bicarbonate <22 compared to group with bicarbonate >28, chi2=30.6197 (p-value <0.0001)							
Mortality in group with bicarbonate 22-28 compared to group with bicarbonate >28, chi2=0.0003 (p-value=0.986)							

When the final cohort was divided into CKD and non-CKD groups (Table 3), the low bicarbonate sub-group had higher in-hospital mortality compared to the normal bicarbonate sub-group in both the CKD (p=0.013) and non-CKD (p<0.001) groups. In comparing the low bicarbonate sub-group to the high bicarbonate sub-group, this difference was statistically significant only in the non-CKD group (p<0.001). For secondary outcomes, in both the CKD and non-CKD groups, more patients with low serum bicarbonate levels required vasopressors and these patients had significantly fewer pressor-free days compared to patients with normal or high bicarbonate levels (p-value<0.001). Regarding other secondary outcomes, only patients in the low bicarbonate sub-group of the non-CKD group had significantly fewer hospital-free days, ICU-free days, and ventilator-free days (p-value<0.001), and no statistically significant difference was seen in the three subgroups with CKD.

**Table 3:** Mortality comparison among different bicarbonate sub-groups in CKD and non-CKD groups CKD: Chronic Kidney Disease

	Patients with history of CKD		Patients with no history of CKD	
	Chi-square	p-value	Chi-square	p-value
Bicarbonate <22 vs 22-28	6.125	0.013	51.971	<0.001
Bicarbonate <22 vs >28	0.986	0.321	31.459	<0.001
Bicarbonate 22-28 vs >28	0.288	0.592	0.032	0.858

Using a multivariate logistic regression model adjusted for age, gender, CKD, COPD, liver cirrhosis, and SOFA score, the primary outcome was compared between group 1 (bicarbonate level <22 mEq/L), group 2 (bicarbonate level 22-28 mEq/L), and group 3 (bicarbonate level >28 mEq/L). As we found a significant interaction between the SOFA score and low bicarbonate, this interaction term was then introduced into the regression model. We found that when adjusted for these variables, the association of low serum bicarbonate with increased in-hospital mortality was seen only when the SOFA score was ≥ 9. When bicarbonate was higher than 28, the mortality rate was higher but not statistically significant (Table 4).

**Table 4:** Estimation results of coefficients for both primary and secondary outcomes CKD: Chronic Kidney Disease, COPD: Chronic Obstructive Lung Disease, SOFA: Sequential Organ Failure Assessment

	Mortality	Hospital-free days	ICU-free days	Ventilator-free days	Pressor-free days
Intercept	-3.57 (<0.001)	3.029 (<0.001)	3.073 (<0.001)	1.046 (<0.001)	2.378 (<0.001)
Age	0.016 (<0.001)	-0.001 (0.127)	0.001 (0.001)	0.002 (0.179)	-0.001 (0.467)
Male	0.006 (0.935)	0.045 (<0.001)	0.010 (0.242)	0.030 (0.471)	0.061 (0.119)
CKD	-0.280 (0.002)	-0.037 (0.013)	-0.007 (0.526)	-0.103 (0.061)	-0.092 (0.049)
COPD	0.139 (0.083)	0.009 (0.488)	0.023 (0.018)	0.074 (0.111)	0.039 (0.355)
Cirrhosis	0.519 (<0.001)	-0.072 (0.013)	0.001 (0.978)	0.028 (0.777)	-0.229 (0.003)
SOFA	0.212 (<0.001)	-0.033 (<0.001)	-0.018 (<0.001)	-0.046 (<0.001)	-0.060 (<0.001)
Group 1	-0.401 (0.069)	0.038 (0.002)	0.141 (<0.001)	0.298 (<0.016)	0.040 (0.355)
Group 3	0.184 (0.117)	-0.082 (<0.001)	-0.056 (<0.001)	-0.117 (0.059)	-0.048 (0.466)
Group 1 SOFA interaction	0.050 (0.017)		-0.013 (<0.001)	-0.025 (0.052)	
n	4176	4176	4176	4176	4176
Log-likelihood	-2330.1	-10116.1	-11893.5	-2970.1	-1765.6

In addition, the interaction term between the low bicarbonate group and the SOFA score was significantly positive, suggesting that the impact of the SOFA score on mortality was higher among patients in group 1. The odds ratio of the SOFA score for the normal bicarbonate group was 1.236 (Table 5), suggesting a 23.6 % increase in the odds of mortality for every unit increase in the SOFA score. The odds ratio of SOFA score for the low bicarbonate group was 1.300 (exp (0.212+0.05) (Table 4), suggesting a 30.0% increase in the odds of mortality for every unit increase in the SOFA score.

**Table 5:** Regression model for primary and secondary outcomes variables CKD: Chronic Kidney Disease, COPD: Chronic Obstructive Lung Disease, SOFA: Sequential Organ Failure Assessment

	Mortality	Hospital-free days	ICU-free days	Ventilator-free days	Pressor-free days
Intercept	0.028	20.672	21.609	2.847	10.781
Age	1.016	0.999	1.001	1.002	0.999
Male	1.006	1.046	1.01	1.031	1.062
CKD	0.756	0.963	0.993	0.902	0.913
COPD	0.87	0.991	0.977	0.928	1.04
Cirrhosis	1.681	0.93	1.001	1.028	0.795
SOFA	1.236	0.968	0.982	0.955	0.942
Group 1	0.67	1.039	1.151	1.347	1.041
Group 3	1.202	0.921	0.945	0.889	0.953
Group 1 SOFA interaction	1.051		0.987	0.975	

The estimation coefficients of the Poisson regression model for hospital-free days are listed in Table 4. The interaction between group 1 and SOFA score was not significant and was not included for further analysis. For the non-zero portion, Group 1 had significantly higher hospital-free days, while group 3 had significantly lower hospital-free days. The incident rate ratio for group 1 was 1.039 and that for group 3 was 0.921.

The estimation coefficients of the Poisson regression model for ICU-free days are listed in Table 4. For the non-zero portion, patients in group 3 (bicarbonate > 28) had significantly fewer ICU-free days (p<0.01). The interaction term of bicarbonate group 1 and SOFA score was significantly negative, suggesting that the difference in ICU-free days between the low bicarbonate group and the normal bicarbonate group would depend on patients’ SOFA score. When the SOFA score was ≥ 11 (0.141/0.013; Table 4), patients in group 1 had fewer ICU-free days. This also suggested that the impact of SOFA score on the ICU-free days was more significant on patients in group 1 compared to group 2. For group 2, for every unit increase in the SOFA score, there was a 1.8 % decrease in ICU-free days (Table 4). For group 1, for every unit increase in the SOFA score, there was a 3.1 % decrease in ICU-free days.

The estimation coefficients of the Beta regression model portion for ventilator-free days are listed in Table 4. The interaction term of bicarbonate group 1 and SOFA score was negative suggesting that the difference in ventilator-free days between group 1 and group 2 was dependent on the SOFA score, although the difference was not statistically significant (p=0.052). When the SOFA score was ≥ 12 (0.298/0.025; Table 4), patients in group 1 had fewer transformed ventilator-free days suggesting that the impact of SOFA score on the ventilator-free days was larger on patients in group 1 compared to group 2. For patients in group 2, with every unit increase in SOFA score, there was a 4.5 % decrease in the average transformed ventilator-free days. For patients in group 1, with every unit increase in SOFA score, there was a 6.9 % decrease in the average transformed ventilator-free days.

The estimation coefficients of the Beta regression model portion for pressor-free days are listed in Table 4. The interaction between group 1 and SOFA score was not significant and was not included for further analysis. We found that there was no significant difference in pressor-free days between the groups.

## Discussion

Sepsis and its complications remain the leading cause of morbidity and mortality worldwide [3,13]. Metabolic acidosis [14,15], elevated lactate [16-20], and acute kidney injury [21-23] are known predictors of worse clinical outcomes in patients with sepsis. While these factors have been extensively investigated for their role in sepsis, the predictive value of serum bicarbonate in sepsis remains unexplored. Recent studies have shown that serum bicarbonate and lactate do not correlate, suggesting that serum bicarbonate cannot be a surrogate for lactate and vice versa [10-12, 24]. Moreover, with a significant proportion of hospitals in rural areas operating without access to rapid lactate levels [25,26], knowing the predictive value of serum bicarbonate in sepsis will always help in risk stratification.

In this retrospective analysis including 4176 patients, we investigated the predictive value of serum bicarbonate in sepsis. Our study suggests that serum bicarbonate level at presentation can predict in-hospital mortality as well as the ICU-free days, ventilator-free days, and hospital-free days. In our unadjusted analysis, serum bicarbonate level < 22 mEq/l had a significant association with elevated in-hospital mortality; however, when controlled for confounders, the difference in the in-hospital mortality was not statistically significant except for when the SOFA score was ≥9. When the SOFA score was ≥9, the mortality rate among patients with serum bicarbonate levels < 22meq/l was significantly higher. Similarly, patients with low serum bicarbonate (< 22 mEq/l) had significantly fewer ICU-free days when the SOFA score was ≥11 and significantly fewer ventilator-free days when the SOFA score was ≥12. In contrast, patients with low serum bicarbonate had significantly more hospital-free days while the patients with high serum bicarbonate had fewer hospital-free days compared to patients with normal serum bicarbonate. It was not clear from this study, however, why the patients with low serum bicarbonate had higher hospital-free days.

An interesting finding of our study was the interaction between SOFA score and serum bicarbonate at presentation suggesting that the predictive value of SOFA score for in-hospital mortality, ICU-free days, ventilator-free days, and hospital-free days was higher among patients with low serum bicarbonate. This brings up a valid question if we should incorporate serum bicarbonate levels into the SOFA score to predict clinical outcomes.

Increasing attention has been dedicated to the utilization of sodium bicarbonate in the treatment of sepsis. Current guidelines [13] recommend against its use based on two small cohort studies [27,28]. However, a few recent studies suggested improved mortality with the administration of sodium bicarbonate in a subset of patients with acute kidney injury [29,30] based on which, a weak recommendation was made in favor of sodium bicarbonate therapy in a subset of patients with septic shock, severe metabolic acidosis (pH ≤ 7.2), and acute kidney injury (AKI) (Acute Kidney Injury Network (AKIN) score 2 or 3). Of note, patients in this study were included if their enrollment SOFA score exceeded 4. Based on our study, low serum bicarbonate had a worse clinical outcome only when the SOFA score was ≥9. With these findings, we propose that the clinical outcomes might be better if the patient population is appropriately chosen with a SOFA score of ≥9 for further studies involving the administration of bicarbonate in patients with sepsis.

Given the retrospective nature of our study, multiple limitations exist. The limitations of our study include the fact that (a) it was a single-center study, (b) the study had a retrospective design, and (c) the reason behind the high bicarbonate level (>28 mEq/L) was not investigated.

## Conclusions

Given the widespread availability of serum bicarbonate levels in health care facilities around the globe, we believe that the utilization of serum bicarbonate levels at presentation along with other markers of sepsis can help in the prognostication of these patients with sepsis. Also noted in our study is that the predictive value of the SOFA score was dependent on the serum bicarbonate level at presentation. Based on this result, we believe that incorporating serum bicarbonate level in the SOFA score might increase the predictive value of the SOFA score. We propose that further prospective studies be performed to evaluate the utilization of serum bicarbonate for these purposes.
